# Altered Resting-State Neural Oscillations and Functional Connectivity in Persistent Postural-Perceptual Dizziness

**DOI:** 10.3390/brainsci16101056

**Published:** 2026-09-30

**Authors:** Feng Li, Chi Zhang, Weiwei Jiang, Bo Song, Yingxin Wang, Lu Tang, Shanshan Chen, Xiangyi Liu, Jingping Shi

**Affiliations:** Department of Neurology, The Affiliated Brain Hospital of Nanjing Medical University, No. 264 Guangzhou Road, Nanjing 210029, China; leefeng1989@163.com (F.L.); zhang_chi2036@163.com (C.Z.); jwwnj@163.com (W.J.); s15667500813@163.com (B.S.); wangyx20081203@126.com (Y.W.); dr.lu.tang@foxmail.com (L.T.); chenshanshan@njmu.edu.cn (S.C.); 18056047013@163.com (X.L.)

**Keywords:** PPPD, electroencephalography, resting state, neural oscillations, functional connectivity

## Abstract

**Highlights:**

**What are the main findings?**

- Multimodal EEG and posturography characterized PPPD-related brain alterations.
- PPPD exhibited frequency-specific changes in EEG power and connectivity.

**What are the implications of the main findings?**

- PPPD may involve abnormal resting-state interactions among cognitive–affective, visual, vestibular-related, and sensorimotor systems.
- Results provide neurophysiological insights into PPPD mechanisms.

**Abstract:**

**Background:** Persistent postural-perceptual dizziness (PPPD) is a chronic functional neuro-otologic disorder marked by persistent dizziness, unsteadiness, and visual dependence. Its electrophysiological mechanisms remain unclear. This study examined resting-state EEG spectral power and functional connectivity in PPPD and their associations with postural stability and clinical symptoms. **Methods:** Forty patients with PPPD and 40 age- and sex-matched healthy controls were studied with an eyes-closed resting-state EEG recording. Static postural stability was measured with the Pro-Kin system. Spectral power was calculated for delta, theta, alpha, beta, and gamma bands. Functional connectivity was assessed using the weighted phase lag index. **Results:** Static posturography showed impaired postural stability in patients with PPPD compared with healthy controls. Spectral power analysis showed increased theta-band relative power over the frontal and left parieto–occipital regions. Connectivity analysis showed increased prefrontal–temporal and decreased temporal–occipital connectivity in the theta band, increased fronto–parieto–occipital and decreased parietal–temporal connectivity in the alpha band, and decreased cerebellar and temporal, central connectivity in the gamma band. **Conclusions:** PPPD was associated with frequency-specific alterations in resting-state cortical oscillations and functional connectivity involving cognitive–affective, visual, and sensorimotor networks. Resting-state EEG may help characterize network-level abnormalities in PPPD.

## 1. Introduction

Persistent postural-perceptual dizziness (PPPD) is a chronic functional neuro-otologic disorder characterized by persistent dizziness, non-spinning vertigo, or unsteadiness lasting for at least 3 months and exacerbated by upright posture, active or passive motion, and exposure to moving or complex visual stimuli [1]. It is increasingly recognized as a common cause of chronic vestibular symptoms in neurology and dizziness clinics and may impose a substantial burden on daily activities, emotional well-being, and health-related quality of life [2,3,4]. Despite standardized diagnostic criteria, PPPD remains clinically challenging because vestibular tests and conventional neuroimaging are often non-specific, whereas symptoms are frequently accompanied by anxiety, depression, visual dependence, and impaired postural control [5,6]. Consequently, objective neurophysiological markers that reflect the underlying network dysfunction are still needed.

Current evidence suggests that PPPD is not solely attributable to peripheral vestibular dysfunction, but may involve maladaptive interactions among vestibular, visual, somatosensory, postural, and affective systems [7,8]. Structural and functional neuroimaging studies have reported abnormalities in multimodal vestibular regions, visual cortices, insular networks, frontal regulatory regions, and cerebellar–sensorimotor circuits [9,10,11,12]. Network-level alterations have also been described in patients with phobic postural vertigo and PPPD, supporting the view that chronic dizziness may be associated with altered cortical integration rather than isolated vestibular end-organ dysfunction [13]. However, most available studies have relied on MRI-based measures, which provide limited temporal resolution for characterizing neural oscillatory dynamics. Although clinical and neurophysiological studies have begun to explore electrophysiological features in PPPD, resting-state EEG evidence remains limited, particularly regarding frequency-specific power changes and phase-based functional connectivity patterns [14]. In addition, few studies have integrated EEG indices with quantitative posturography in the same cohort [15].

The present study was designed to investigate resting-state EEG abnormalities in patients with PPPD using spectral power analysis and weighted phase lag index-based functional connectivity. We hypothesize that PPPD would be associated with frequency-specific alterations in cortical oscillatory activity and functional connectivity involving prefrontal, visual, temporal, cerebellar, and sensorimotor regions. We further propose that these EEG alterations would be related to dizziness-related disability and postural instability. To test these hypotheses, patients with PPPD and age- and sex-matched healthy controls were assessed using clinical scales, static posturography, and 71-channel resting-state EEG. This study aimed to clarify whether resting-state electrophysiological markers may help characterize the neural network alterations associated with PPPD and provide a basis for future mechanistic and longitudinal investigations.

## 2. Materials and Methods

### 2.1. Participants

This study enrolled 40 patients with persistent postural-perceptual dizziness (PPPD) who were recruited from the outpatient and inpatient services of Nanjing Brain Hospital between April 2025 and April 2026. The group assessment was conducted by two experienced neuro-otologists who evaluated independently. The PPPD group included 15 men and 25 women, with a mean age of 58.58 ± 9.76 years. All patients met the 2017 Bárány Society diagnostic criteria for PPPD. The inclusion criteria were as follows: (1) age between 30 and 75 years; (2) absence of symptoms and signs of cerebellar impairment; and absence of other neurological disorders, including traumatic brain injury, intracranial tumors, or cerebrovascular disease; (3) dizziness caused by other recent vestibular disorders, such as benign paroxysmal positional vertigo, Ménière’s disease, sequelae of vestibular neuritis, vestibular migraine; (4) no use of medications that could affect resting-state brain activity, such as antidepressants, anxiolytics, or antiepileptic drugs, within 4 weeks before enrollment.

Forty age- and sex-matched healthy controls were recruited. Control participants had no history of otologic, neurological, or psychiatric disorders and had not used medications known to affect resting-state brain activity. The study protocol was approved by the Ethics Committee of Nanjing Medical University Affiliated Brain Hospital (Approval No: IRB-AF40-1.0). Written informed consent was obtained from all participants before study participation.

### 2.2. Clinical Assessment

Demographic and clinical data were collected before EEG acquisition. The recorded variables included sex, age, and disease duration. Dizziness severity and balance-related symptoms were assessed using the Visual Analog Scale (VAS), Dizziness Handicap Inventory (DHI) [16,17], and Activities-specific Balance Confidence Scale (ABC) [18]. Psychological symptoms were evaluated using the Hamilton Anxiety Rating Scale (HAMA) and Hamilton Depression Rating Scale (HAMD).

### 2.3. Static Posturography

**Figure 1:**
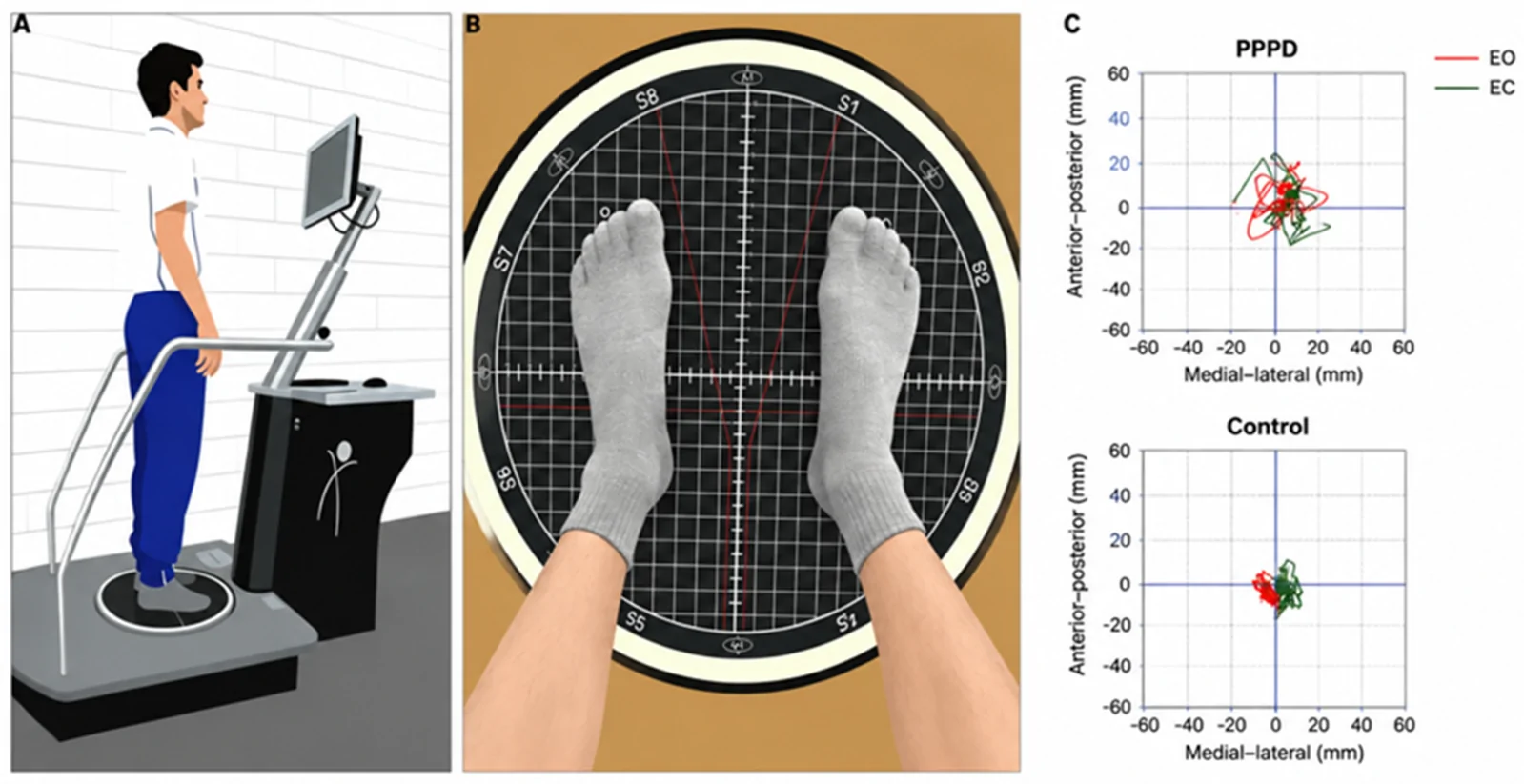
Balance assessment using Pro-Kin visual feedback balance system. (**A**) Participants stood on a force platform. (**B**) The platform recorded center-of-pressure (COP) data during participants standing with eyes open (EO) and eyes closed (EC). (**C**) Representative COP sway trajectories in a patient with persistent postural-perceptual dizziness (PPPD) and a healthy control. The PPPD showed a larger sway area and greater excursions compared with the control.

Static postural stability was assessed using the Pro-Kin visual feedback balance system (PK254; TecnoBody S.R.L., Bergamo, Italy) [19]. Participants were instructed to stand comfortably on the force platform in a standardized position, with their arms placed alongside their bodies and their gaze fixed on a target located directly in front of them. Each participant completed two 30 s trials under eyes-open (EO) and eyes-closed (EC) conditions. Center-of-pressure (COP) data were recorded using pressure-sensitive sensors. The following posturographic parameters were analyzed: COP trajectory length, ellipse sway area, and mean sway velocity in the anterior–posterior and medio-lateral directions. Greater trajectory length, larger sway area, and higher sway velocity were interpreted as indicators of poorer postural stability. See Figure 1 for further details on static posturography.

### 2.4. EEG Acquisition

Resting-state EEG was recorded using a 71-channel whole-head EEG cap system (BoruiKang Technologies, Changzhou, China) [20]. The electrode montage covered conventional cortical regions and cerebellar electrodes, including PO9, PO10, O9, O10, Iz, CBz, CB1, and CB2. Electrode placement followed the international 10–10 system. The reference electrode was positioned at CPz, and the ground electrode was positioned at AFz.

EEG signals were sampled at 1000 Hz. Electrode impedance was maintained below 20 kΩ throughout acquisition. Recordings were performed during the daytime in a quiet environment. Participants were asked to wash their scalp before testing and were screened to ensure the absence of hunger, hypoglycemia, fatigue, or other discomfort. During recording, participants sat quietly with their eyes closed, remained awake, relaxed their facial and body muscles, and minimized horizontal and vertical eye movements. A 10 min eyes-closed resting-state EEG recording was obtained from each participant.

Preprocessing was performed using MATLAB R2023a and the EEGLAB v14.1.2 toolbox. Raw EEG data were high-pass filtered at 0.1 Hz and low-pass filtered at 50 Hz. The continuous EEG data were then segmented into 2 s epochs. Epochs containing obvious noise were manually inspected and rejected, and bad channels were interpolated using spherical spline interpolation (see Supplementary Table S1 for more details). Finally, independent component analysis (ICA) was applied to remove artifact components such as eye movements, blinks, and muscle activity, and the data were re-referenced to the whole-brain average reference.

### 2.5. Spectral Power Analysis

Spectral power analysis was performed in MATLAB [21]. The Welch method was applied to resting-state EEG data using a Hamming window, with 50% overlap between adjacent segments. The segment length was set to one-eighth of the total data length. Absolute power was calculated for the following frequency bands: delta, 1–4 Hz; theta, 4–8 Hz; alpha, 8–13 Hz; beta, 13–30 Hz; and gamma, 30–50 Hz. Relative power was then calculated for each frequency band. Cluster-based permutation testing was used to assess between-group differences in spectral power while controlling for multiple comparisons across electrodes [22].

### 2.6. Functional Connectivity Analysis

Functional connectivity features were quantified using the weighted phase lag index (wPLI). The wPLI is an analytical metric based on the phase relationships of EEG signals and is used to measure the degree of phase coupling of neural oscillations between different brain regions, thereby reflecting interregional information transmission and functional connectivity status. wPLI values range from 0 to 1, where 0 indicates no phase coupling and 1 indicates complete phase synchronization; the stronger the coupling between neural oscillations, the greater the value. The wPLI is highly robust against volume conduction effects, random noise, and spurious synchronization [23,24]. Based on these properties, the wPLI can be used to assess the frequency specificity of interactions between different cortical regions, thereby characterizing communication within neural networks across different frequency bands. The four electrodes TP9, TP10, FP1, and FP2 were excluded because they were substantially affected by muscle potentials. For each participant, 67 × 67 functional connectivity matrices were constructed for the delta, theta, alpha, beta, and gamma frequency bands. FIR band-pass filtering (using the firls least squares filter) was applied to the continuous or segmented EEG data of each subject, with the filtering range being 1–4 Hz, 4–8 Hz, 8–13 Hz, 13–30 Hz, and 30–50 Hz. To avoid filtering edge effects, the data before and after the filtering of each channel were removed by 10%. Hilbert transformation was performed on the filtered signals of each channel to obtain the analytic signals, and the cross-spectrum between each electrode pair was calculated. The imaginary part of the cross-spectrum was used for wPLI estimation. Mean wPLI values were then calculated within each frequency band and compared between groups.

### 2.7. Statistical Analysis

Statistical analyses were performed using SPSS version 25.0 (SPSS Inc., Chicago, IL, USA) and MATLAB. Normality and homogeneity of variance were examined before group comparisons. Continuous clinical variables were compared using independent-samples t tests when parametric assumptions were satisfied. For EEG spectral power and functional connectivity analyses, between-group differences were assessed across frequency bands. Cluster-based permutation statistics were applied to spectral power analyses to control for multiple comparisons across EEG electrodes. Independent-samples t-statistics for between-group differences were calculated at each electrode. A two-sided uncorrected *p* < 0.05 was used as the cluster-forming threshold. Samples exceeding this threshold that were spatially adjacent and, where applicable, temporally contiguous in the same direction were combined into a cluster. Electrode adjacency was predefined based on the actual spatial coordinates of the 71 electrodes. In each permutation, the maximum absolute cluster statistic across the five frequency bands (delta, theta, alpha, beta, and gamma) was retained, thereby simultaneously correcting for multiple comparisons across frequency bands and electrodes. A two-sided cluster-level corrected *p* < 0.05 was considered statistically significant. For Functional connectivity, Network-Based Statistics (NBS) were applied to compare wPLI matrices between PPPD groups and healthy controls [25]. NBS testing was performed using the NBS1.2 toolbox in MATLAB R2023a, with the primary threshold set at *p* < 0.001. The overall family-wise error (FWE) rate control threshold was set at *p* < 0.05. Network FC strength was defined as the mean FC of all edges within the network. Brain network visualization was performed using BrainNet Viewer. Because HAMA and HAMD scores were significantly higher in the PPPD group than in the HC group, to control for the potential confounding effects of affective symptoms, analysis of covariance (ANCOVA) with HAMA and HAMD scores as covariates was used to compare EEG spectral power and functional connectivity between the two groups. Static posturographic outcomes were analyzed using separate two-way mixed-design repeated-measures analyses of variance (ANOVAs), with group (PPPD vs. HC) as the between-subject factor and visual condition (EO vs. EC) as the within-subject factor. In the PPPD group, correlation analyses were performed between spectral power showing significant differences and clinical characteristics, balance assessments, and psychological scale scores. Correlation analyses were also performed between brain functional connectivity showing significant differences and static balance parameters. Pearson correlation analysis was used if the data were normally distributed. False discovery rate (FDR) correction was also applied. FDR correction was performed using the Benjamini–Hochberg method. All comparisons were two-tailed, and the level of statistical significance was set at *p* < 0.05.

## 3. Results

### 3.1. Clinical Characteristics

Demographic and clinical characteristics of the subjects are summarized in Table 1. No significant between-group differences were observed in age and sex distribution. Compared with healthy controls, patients with PPPD had significantly higher HAMA and HAMD scores, indicating greater anxiety and depressive symptom burden.

### 3.2. Static Postural Stability

Static posturography showed impaired postural stability in patients with PPPD compared with healthy controls. Detailed statistical analyses of all parameters were shown in Table 2. Under the eyes-open condition, patients with PPPD showed significantly greater medio-lateral average sway speed (*p* = 0.002), anterior–posterior average sway speed (*p* = 0.003), and trajectory area (*p* = 0.024). COP trajectory length under the eyes-open condition showed an increasing trend but did not reach statistical significance (*p* = 0.056).

Under the eyes-closed condition, patients with PPPD showed significantly greater medio-lateral average sway speed (*p* = 0.020), anterior–posterior average sway speed (*p* = 0.002), and trajectory area (*p* = 0.030) than healthy controls. COP trajectory length under the eyes-closed condition showed an increasing trend but did not reach statistical significance (*p* = 0.066).

The mixed-design analyses showed significant main effects of visual condition for all outcomes (*p* < 0.001), with higher values under EC than EO. Significant main effects of group were observed for medio-lateral average sway speed (F(1, 78) = 7.775, *p* = 0.007, η^2^ = 0.827), anterior–posterior average sway speed (F(1, 78) = 11.473, *p* = 0.001, η^2^ = 0.685), and sway area (F(1, 78) = 5.280, *p* = 0.024, η^2^ = 0.847), but not for trajectory length (F(1, 78) = 391.565, *p* = 0.052, η^2^ = 0.834). A significant group-by-visual-condition interaction was detected only for anterior–posterior average sway speed (F(1, 78) = 4.664, *p* = 0.034, η^2^ = 0.056). Simple-effects analyses showed that anterior–posterior average sway speed increased from EO to EC in both groups (PPPD: t = 9.552, *p* < 0.001; HC: t = 8.928, *p* < 0.001) and was greater in the PPPD group under both EO and EC conditions (both *p* ≤ 0.003).

### 3.3. Resting-State EEG Spectral Power

**Figure 2:**
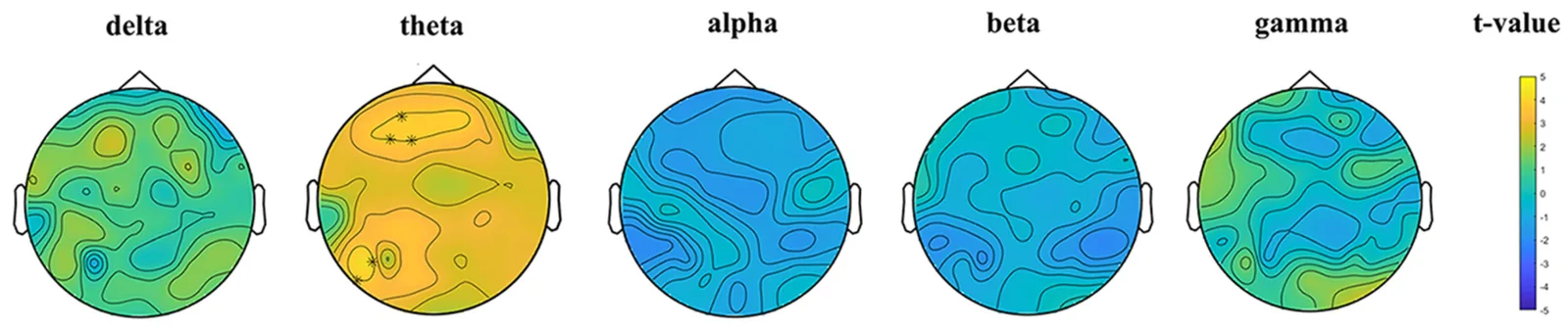
Topographic maps of t-values for EEG power spectrum differences between PPPD patients and healthy controls across five frequency bands. The t-value topographies illustrate the statistical differences in power spectral density (PSD) between the PPPD group and healthy controls in the delta (1–4 Hz), theta (4–8 Hz), alpha (8–13 Hz), beta (13–30 Hz), and gamma (30–50 Hz) bands. Black asterisks denote scalp regions showing statistically significant between-group differences (*p* < 0.05, corrected).

Cluster-based permutation analysis revealed significant between-group differences in theta-band relative power (Figure 2). Compared with healthy controls, patients with PPPD showed increased theta-band power over the frontal region (electrodes: AF3, F1, F3) and left parieto–occipital region (electrodes: PO7, PO9) after controlling for psychiatric comorbidities. No significant between-group differences were detected in delta, alpha, beta, or gamma power.

### 3.4. Functional Connectivity

**Figure 3:**
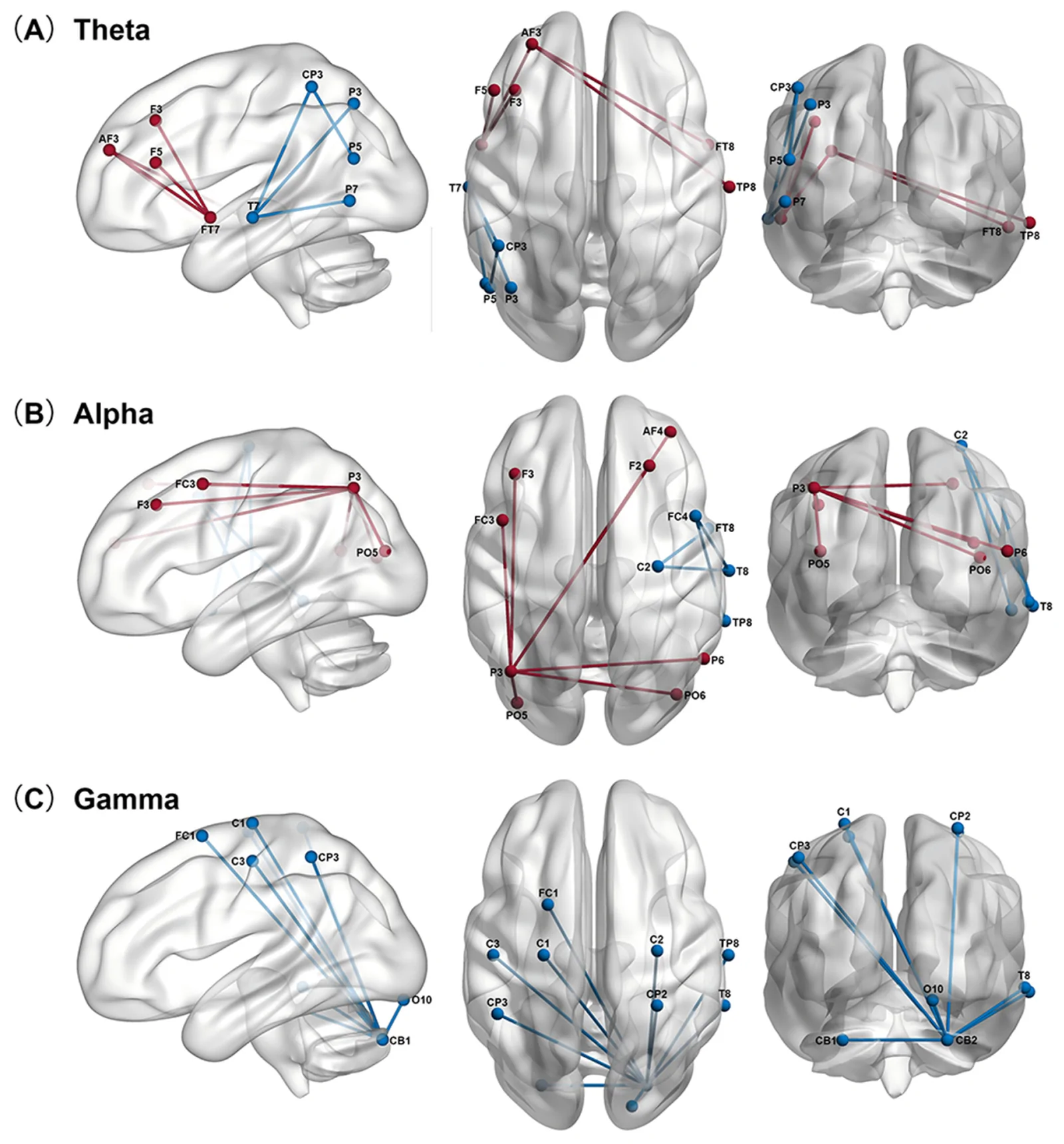
Functional connectivity differences between PPPD patients and healthy controls across frequency bands. Significant between-group differences in functional connectivity are shown for the theta (**A**), alpha (**B**), and gamma (**C**) frequency bands. Red lines indicate significantly increased functional connectivity in patients with PPPD compared to healthy controls, while blue lines indicate significantly decreased functional connectivity. Labeled nodes denote the electrode sites involved in the significant connections. Electrode abbreviations: AF: anterior frontal; F: frontal; FC: frontocentral; C: central; CP: centroparietal; P: parietal; PO: parieto–occipital; O: occipital; T: temporal; TP: temporoparietal; CB: cerebellar.

wPLI-based functional connectivity analysis showed frequency-specific alterations in patients with PPPD compared to healthy controls (Figure 3). After controlling for psychiatric comorbidities, temporal–occipital connectivity was decreased, whereas prefrontal–temporal connectivity was increased in the theta band. Connectivity among frontal, parietal, and occipital regions was increased, and parietal–temporal connectivity was decreased in the alpha band. Cerebellar and temporal, central connectivity was decreased in the gamma band. No significant differences were observed in cerebellar-related connectivity in the remaining frequency bands.

### 3.5. Correlation Analysis

**Figure 4:**
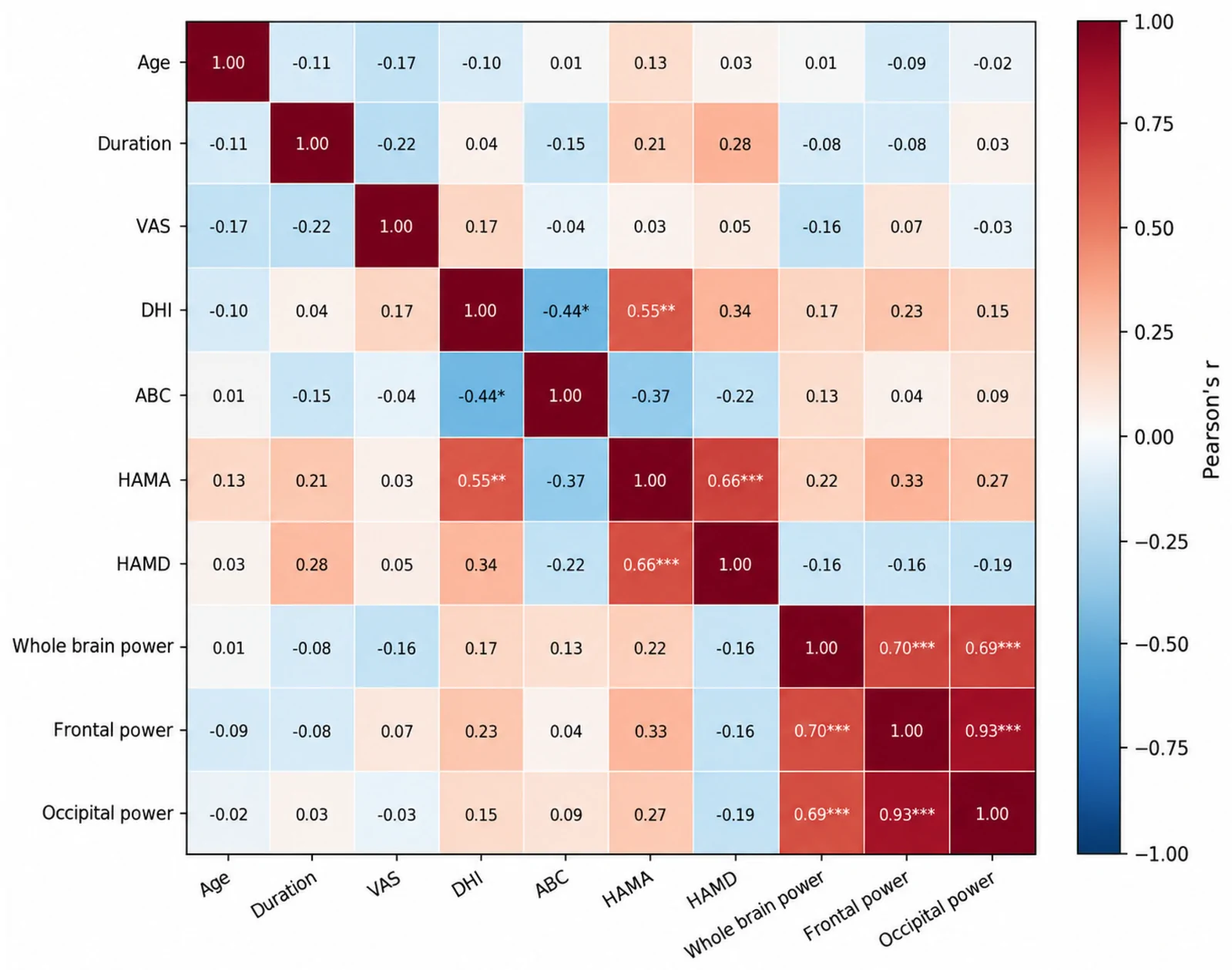
Correlation analysis between clinical assessments and EEG spectral power in patients with PPPD (N = 40). Red indicates positive correlation, blue indicates negative correlation. Asterisks indicate statistical significance after Benjamini–Hochberg false discovery rate correction: * q < 0.05, ** q < 0.01, *** q < 0.001. ABC, Activities-specific Balance Confidence Scale; DHI, Dizziness Handicap Inventory; HAMA, Hamilton Anxiety Rating Scale; HAMD, Hamilton Depression Rating Scale.

Correlation analyses were performed within the PPPD group (Figure 4). Pearson correlation analysis was performed to examine the associations among clinical variables and EEG power, brain functional connectivity and static stability parameters in patients with PPPD. To account for multiple comparisons, false discovery rate correction was applied using the Benjamini–Hochberg procedure. A total of 45 pairwise correlations were tested across 10 numerical variables.

After FDR correction, frontal power was positively correlated with occipital power (r = 0.930, *p* < 0.001, FDR-adjusted q < 0.001). HAMA scores were positively correlated with HAMD scores (r = 0.658, *p* < 0.001, q < 0.001). DHI scores were positively correlated with HAMA scores (r = 0.547, *p* < 0.001, q = 0.0024). In addition, DHI scores were negatively correlated with ABC scores (r = −0.441, *p* = 0.004, q = 0.033). HAMA scores and frontal power reached nominal statistical significance before correction but did not survive FDR correction (r = 0.332, *p* = 0.036, q = 0.182).

Pearson correlation analyses were also performed to examine the associations between two functional connectivity measures and posturographic parameters under eyes-open (EO) and eyes-closed (EC) conditions. The two connectivity measures included theta-band temporal–occipital functional connectivity and prefrontal–temporal functional connectivity. A total of 16 correlations were tested, and multiple comparisons were controlled using the Benjamini–Hochberg false discovery rate (FDR) procedure. After FDR correction, none of the correlations between functional connectivity and static stability parameters were significant (all q > 0.05).

## 4. Discussion

In this study, PPPD was associated with frequency-specific resting-state EEG alterations, including increased theta-band relative power over frontal and left parieto–occipital regions, altered theta-, alpha-, and gamma-band functional connectivity, and impaired postural stability. These findings support the presence of altered electrophysiological patterns involving cognitive–affective, visual, and sensorimotor systems in PPPD, while requiring confirmation in larger longitudinal cohorts.

Theta oscillations have been implicated in long-range communication involved in cognitive control and contextual integration, particularly in fronto–temporal and fronto–parietal systems [26]. However, vigilance may have contributed to the observed theta-band differences. The theta findings should be interpreted cautiously, without attributing them specifically to PPPD-related attentional or affective mechanisms. The power of the frontal and parietal lobes was highly correlated among the participants (r = 0.93), indicating significant spectral consistency. This finding suggests that the observed power differences partly reflect the overall contribution rather than independent regional variations. Additionally, the correlation between HAMA scores and frontal power did not survive after FDR correction; the current analysis cannot determine regional specificity. Patients with PPPD had significantly higher HAMA and HAMD scores; this may be linked to emotional burden in PPPD. Affective symptoms, particularly anxiety and depression, are highly prevalent in patients with PPPD and are increasingly recognized as important modulators of both symptom severity and central nervous system functioning. Recent clinical studies have shown that anxiety-related hypervigilance, fear of movement, and maladaptive threat monitoring may contribute to persistent dizziness and postural instability in PPPD, forming part of a self-reinforcing perceptual–affective loop rather than representing purely comorbid psychiatric conditions [27,28,29,30]. In the present study, ANCOVA results demonstrated that after controlling for HAMA and HAMD scores, several EEG abnormalities—particularly posterior theta power and specific theta/alpha/gamma connectivity alterations—remained significant. HAMA and HAMD scores differed substantially between groups, limiting the separation of group-related and affective-symptom-related variation. Covariate-adjusted estimates depend on the model and the available overlap in symptom scores and do not establish that the EEG findings are specific to PPPD or independent of affective symptoms.

The occipital and left parieto–occipital theta-band findings are also consistent with previous neuroimaging evidence implicating visual and posterior cortical regions in PPPD. Li et al. reported altered spontaneous activity in the right precuneus and cuneus in patients with PPPD, indicating that posterior cortical regions involved in visuospatial processing may contribute to the pathophysiology of this disorder [10]. In the present study, increased theta power in the occipital/parieto–occipital region and decreased temporal–occipital connectivity may indicate altered top-down modulation of visual processing during rest. This interpretation is also consistent with studies of visually induced dizziness, in which altered functional brain connectivity has been observed in networks related to visual motion perception and multisensory integration [31,32]. Nevertheless, because EEG source localization was not performed, the anatomical interpretation of scalp-level occipital and parieto–occipital signals should remain cautious.

The decreased theta-band temporal–occipital connectivity and alpha-band parietal–temporal connectivity may reflect impaired integration between vestibular-related, auditory/temporal, visual, and higher-order regulatory systems [33,34]. Previous fMRI work in chronic subjective dizziness demonstrated altered activity in vestibular and insular systems during vestibular stimulation, suggesting that abnormal processing within vestibular–interoceptive networks may contribute to chronic dizziness symptoms [35]. Moreover, Li et al. reported altered intra- and inter-network functional connectivity in PPPD, including abnormal interactions between visual, sensorimotor, and default-mode-related networks [9]. The present EEG findings extend these observations by showing that such network alterations may be frequency-specific and detectable in resting-state electrophysiological connectivity.

The increased fronto–parieto–occipital connectivity in the alpha band may be related to enhanced visual–spatial monitoring or increased reliance on visual information for postural control [36,37]. This interpretation is supported by the posturographic findings: patients with PPPD showed significantly greater COP displacement than controls. Similar behavioral observations have been reported in patients with phobic postural vertigo, in whom gait and postural control were influenced by fear of falling, attention, and visual input [38]. Experimental work has also shown that visual motion can modulate postural sway, supporting the role of visual input in balance control [39]. Together with the present EEG findings, these data suggest that excessive visual weighting and altered cortical control of sensory integration may participate in PPPD-related postural instability. However, the present study did not directly manipulate visual motion or vestibular stimulation; therefore, visual dependence was inferred from postural performance rather than experimentally tested.

In the gamma band, decreased connectivity between cerebellar and temporal, central may be clinically relevant because cerebellar and sensorimotor networks are important for postural control, movement calibration, and vestibular–motor integration. In recent years, with the advancement of recording and analysis technologies, the successful capture of cerebellar electrical activity has become possible [40,41]. Prior multimodal imaging work in phobic postural vertigo reported cortical and cerebellar alterations, suggesting that distributed motor and vestibular networks may be involved in functional dizziness syndromes [12]. In addition, structural connectome studies have demonstrated that the vestibular cortical network is widely distributed and includes multimodal sensory and motor-related regions [42]. In the previous research, error-related potentials (ErrP) in the cerebellar scalp region were induced and recorded through an error judgment experiment, and the introduction of cerebellar region features enhanced the online classification effect of ErrP [43]. This proved the reliability of cerebellar activity. However, this should be interpreted cautiously; gamma-band EEG is susceptible to muscle and other non-neural artifacts, and scalp-level EEG has limited ability to localize cerebellar generators. Without an adequate artifact-sensitivity analysis and source-level validation, these findings cannot be attributed to cerebellar generators or interpreted as evidence of cerebellar dysfunction.

No significant beta-band difference was identified in the present study, whereas Kim et al. recently reported increased high-beta activity in PPPD [44]. This discrepancy may principally reflect methodological differences. The studies differed in frequency range, number of electrodes, sample size, reference montage, electrode density, recording posture, preprocessing, and clinical design.

The main contribution of this study is that regional spectral power, wPLI-based connectivity, posturography, and psychological measures were examined within the same cohort. These findings indicate that PPPD is associated with selective, frequency-dependent reorganization of functional brain networks involving posterior sensory regions, frontal control systems, and cerebellar–sensorimotor circuits. Functional connectivity studies have shown that PPPD is not confined to a single abnormal brain region. Lee et al. described altered resting-state connectivity between vestibular-related regions and other sensory and spatial processing regions; Li et al., using network analysis, further observed abnormalities in both within-network and between-network connectivity [9,33]. Studies of visually induced dizziness have also reported functional connectivity reorganization [32]. Based on current evidence, what appears more reproducible than the consistent strengthening or weakening of any single connection is the overall trend toward disrupted coordination among the visual, vestibular, and sensorimotor systems. This pattern aligns with contemporary models of PPPD emphasizing abnormal sensory reweighting and predictive processing dysfunction across distributed cortical and subcortical networks [5,6].

Several limitations should be acknowledged. First, the sample size was modest, with 40 patients and 40 controls, which may limit statistical power and the generalizability of the findings. Second, this was a single-center study, and potential center-specific recruitment or assessment bias cannot be excluded. Third, the cross-sectional design precluded causal inference regarding whether the observed EEG abnormalities represent predisposing factors, compensatory changes, or consequences of persistent dizziness. Fourth, no intervention or longitudinal follow-up was included; therefore, the sensitivity of the identified EEG markers to treatment response remains unknown. Fifth, EEG was recorded only in the eyes-closed resting state, and task-based EEG during visual motion, vestibular stimulation, or postural challenge was not performed. Sixth, scalp-level EEG has limited spatial resolution, and the present findings should not be interpreted as precise localization of deep or cerebellar generators without source-level validation.

## 5. Conclusions

Patients with PPPD showed increased theta-band power over frontal and parieto–occipital regions, frequency-specific alterations in functional connectivity, and impaired postural stability. These findings suggest that PPPD may involve abnormal resting-state interactions among cognitive–affective, visual, vestibular-related, and sensorimotor systems. Future studies should use larger multicenter cohorts, longitudinal designs, source-localized EEG or multimodal EEG–fMRI approaches, and task paradigms involving visual motion or vestibular stimulation. More advanced methods such as graph theory, information theory and computational modeling could provide deeper mechanistic insights into the changes in brain dynamics within the PPPD [45]. Interventional studies, including vestibular rehabilitation, cognitive-behavioral therapy, neuromodulation, or comorbidity treatment such as vestibular migraine [46], are also needed to determine whether these electrophysiological alterations are modifiable and whether they can serve as biomarkers for prognosis or treatment response.

## Figures and Tables

**Table 1:** Clinical characteristics and evaluation of subjects.

	PPPD	Control	*p*
Female/male	25/15	25/15	NS
Age (year)	58.58 ± 9.76	58.12 ± 9.23	NS
Duration (month)	24.13 ± 22.33	-	
VAS	4.88 ± 1.40	-	
DHI	49.05 ± 15.58	-	
ABC	59.20 ± 17.93	-	
HAMA	14.90 ± 4.65	3.72 ± 2.67	<0.001
HAMD	10.75 ± 3.96	2.88 ± 2.14	<0.001

VAS: Visual Analog Scale; DHI: Dizziness Handicap Inventory; ABC: Activities-specific Balance Confidence Scale; HAMA: Hamilton Anxiety Rating Scale; HAMD: Hamilton Depression Rating Scale; NS: not significant.

**Table 2:** Comparison of postural sway parameters between PPPD patients and healthy controls.

Parameter	PPPD	Control	*p*-Value
EO			
ML average sway speed (mm/s)	10.18 ± 2.58	8.38 ± 2.44	0.002
AP average sway speed (mm/s)	7.98 ± 2.24	6.68 ± 1.49	0.003
Trajectory area (mm^2^)	414.25 ± 109.73	361.08 ± 96.69	0.024
Trajectory length (mm)	382.58 ± 108.32	339.82 ± 87.49	0.056
EC			
ML average sway speed (mm/s)	16.42 ± 5.14	14.05 ± 3.67	0.020
AP average sway speed (mm/s)	11.58 ± 3.66	9.25 ± 2.61	0.002
Trajectory area (mm^2^)	813.62 ± 262.50	695.35 ± 214.29	0.030
Trajectory length (mm)	749.22 ± 242.36	656.82 ± 199.41	0.066

Data are presented as mean ± standard deviation. PPPD: persistent postural-perceptual dizziness; EO: eyes-open condition; EC: eyes-closed condition; AP: anterior–posterior direction; ML: medial-lateral direction.

## Data Availability

Due to privacy restrictions regarding sensitive information, we cannot disclose the data used in this study.

## Supplementary Materials

The following supporting information can be downloaded at https://www.mdpi.com/article/10.3390/brainsci16101056/s1, Table S1: EEG Preprocessing parameter.

## Abbreviations

The following abbreviations are used in this manuscript:

PPPD	Persistent Postural-Perceptual Dizziness
EEG	Electroencephalography
rs-EEG	Resting-State Electroencephalography
FC	Functional Connectivity
wPLI	Weighted Phase Lag Index
DHI	Dizziness Handicap Inventory
ABC	Activities-specific Balance Confidence Scale
